# Neural Epidermal Growth Factor-Like 1 (NELL-1)-Associated Membranous Nephropathy: A Clinical and Outcome-Based Study From a Tertiary Care Center

**DOI:** 10.7759/cureus.92016

**Published:** 2025-09-10

**Authors:** Muzamil Ahmad Wani, Shahnawaz Hassan, Amir Farooq, Imtiyaz Wani, Muzafar Maqsood Wani, Rayees Yousuf, Manzoor Parry, Mohammad Ashraf Bhat, Mukaresh Fatima, Mehraj Ul Islam

**Affiliations:** 1 Nephrology, Sher-i-Kashmir Institute of Medical Sciences, Srinagar, IND; 2 Internal Medicine, Sher-i-Kashmir Institute of Medical Sciences, Srinagar, IND

**Keywords:** adult nephrotic syndrome, glomerular disorders, kidney disease, membranous nephropathy, nell-1 antigen

## Abstract

Introduction: Membranous nephropathy (MN) is a leading cause of nephrotic syndrome in adults, with neural epidermal growth factor-like 1 (NELL-1) identified as a novel target antigen in a subset of cases. This study aimed to evaluate the clinical profile, secondary associations, treatment, and outcomes of NELL-1-associated MN in a single-center cohort.

Methods: We conducted a retrospective observational study at a tertiary care center, reviewing all patients diagnosed with MN between January 2022 and April 2024. Patients with NELL-1-positive MN on renal biopsy immunohistochemistry were included. Clinical data, secondary causes (autoimmune diseases, toxic exposures, malignancies), treatments, and outcomes (remission status, proteinuria, serum albumin) were analyzed.

Results: Among 65 MN patients, 12 (18.5%) had NELL-1-associated MN (66.7% female; median age 34 years). Secondary associations included exposure to mercury-containing skin-whitening cream in three patients (25%), malignancy in two (16.7%), and psoriasis in one (8.3%), while six patients (50%) had primary (idiopathic) NELL-1-associated MN. Initial therapy was conservative (renin-angiotensin system blockade and supportive care) in most; seven patients ultimately required immunosuppressive therapy due to persistent nephrotic syndrome or secondary causes. Over a mean follow-up of 22.9 ± 10.6 months, proteinuria significantly decreased (from 5.7 ± 4.3 g/day to 0.5 ± 0.7 g/day; p = 0.002), and serum albumin improved (2.7 ± 1.0 g/dL to 4.0 ± 0.5 g/dL; p = 0.006). By the last follow-up, eight patients (66.7%) achieved complete remission and three (25%) partial remission; one patient died during follow-up.

Conclusion: NELL-1-associated MN encompasses a heterogeneous spectrum of primary and secondary cases. Identifying inciting factors such as toxic exposures or malignancies is crucial, as withdrawal or treatment of the underlying cause can lead to remission. A majority of patients achieved remission with supportive care and/or immunosuppression, but relapses can occur. Long-term follow-up and patient education are essential. Larger studies are needed to inform optimal management strategies for NELL-1-associated MN.

## Introduction

Membranous nephropathy (MN) is a glomerular disease characterized by the thickening of the capillary wall due to subepithelial immune complex deposition. It is among the most common causes of nephrotic syndrome in non-diabetic adults, accounting for approximately 30% of such cases. Since the discovery of the podocyte antigen phospholipase A₂ receptor (PLA₂R) and its association with MN, several additional podocyte antigens have been identified, including thrombospondin type-1 domain-containing 7A (THSD7A), neural epidermal growth factor-like 1 (NELL-1), semaphorin 3B (SEMA3B), and exostosin 1/2 (EXT1, EXT2). Approximately 70% of patients with primary MN have circulating immunoglobulin G (IgG) autoantibodies against PLA₂R [1]. The identification of specific podocyte antigens has enabled an antigen-based framework for the classification, diagnosis, and monitoring of MN.

NELL-1 has been recognized as a target antigen in both primary and secondary MN [2]. NELL-1-associated MN has been linked to malignancies, autoimmune diseases, and thiol-containing medications (e.g., lipoic acid, bucillamine, tiopronin), as well as traditional remedies with high mercury content [2]. It has also been reported as a cause of de novo MN following kidney transplantation [3]. NELL-1 positivity is detected in approximately 16% of PLA₂R-negative MN cases [4], and around 10% of all MN biopsies demonstrate NELL-1 positivity [5]. Global data on NELL-1-associated MN are steadily increasing, and the present study adds to this growing body of literature [6].

## Materials and methods

This retrospective, single-center, observational study was conducted from January 2022 to April 2024 in the Department of Nephrology at Sher-i-Kashmir Institute of Medical Sciences (SKIMS), Srinagar, India, after obtaining approval from the institute's Institutional Ethics Committee (approval number: IEC/SKIMS Protocol # 319/2022). Written informed consent was obtained from all patients. Descriptive statistics were used to summarize the data. Categorical variables (e.g., sex, comorbidities) are presented as frequencies and percentages, while continuous variables (e.g., age, serum creatinine, albumin) are summarized as mean ± standard deviation or median with interquartile range, as appropriate. All renal biopsy specimens were evaluated by an experienced renal pathologist. Biopsies diagnosed as MN by light microscopy, with confirmation by immunofluorescence and electron microscopy, were reviewed. Immunohistochemical staining for PLA₂R was performed for all cases; if PLA₂R was negative, additional staining for THSD7A and NELL-1 was performed.

Patients with NELL-1-positive staining on biopsy were identified as having NELL-1-associated MN and were included in the study. We retrospectively reviewed the clinical records of these patients to identify potential secondary causes, including autoimmune conditions such as systemic lupus erythematosus (SLE), history of skin-whitening agent use or traditional medicines, hepatitis B and C serologies, and age-appropriate malignancy screening.

Initial management for all patients consisted of nonimmunosuppressive antiproteinuric treatment (NIAT), which included blood pressure control, renin-angiotensin system (RAS) blockade, diuretics, sodium-glucose cotransporter-2 (SGLT2), and other supportive measures. Immunosuppressive therapy (IST) was initiated in patients who failed to show an adequate response to NIAT during follow-up or who had an indication for early immunosuppression (e.g., severe secondary disease).

Follow-up data, including 24-hour proteinuria, serum albumin, and estimated glomerular filtration rate (eGFR), were recorded at defined intervals to assess treatment response and disease course. Nephrotic syndrome was defined as proteinuria >3.5 g/day with serum albumin <3.0 g/dL. Complete remission (CR) was defined as proteinuria <0.3 g/day with the normalization of serum albumin and renal function. Partial remission (PR) was defined as a >50% reduction in proteinuria from baseline to a value between 0.3 g/day and 3.5 g/day, with the normalization of serum albumin and stable renal function. No remission was defined as a <50% reduction in proteinuria or persistent nephrotic-range proteinuria (>3.5 g/day). eGFR was calculated using the 2021 Chronic Kidney Disease Epidemiology Collaboration (CKD-EPI) creatinine equation.

## Results

Between January 2022 and April 2024, a total of 65 patients were diagnosed with MN. Of these, 12 patients (18.5%) were identified as having NELL-1-associated MN. Among this cohort, eight patients (66.7%) were female, and the median age was 34 years (IQR: 27-45). Three patients (25%) had a history of using mercury-containing skin-whitening creams, including one patient with concurrent transverse myelitis and SLE. Six patients (50%) had no identifiable secondary cause (primary NELL-1-associated MN). Two patients (16.7%) had underlying tumors, one with a benign ovarian teratoma and the other with a malignant lung carcinoma. One patient (8.3%) had psoriasis. The baseline clinical and laboratory characteristics of the 12 patients are summarized in Table 1. 

**Table 1:** Baseline characteristics of patients with NELL-1-associated MN NELL-1: neural epidermal growth factor-like 1

Characteristic	Value
Age in years (mean ± SD)	37.25 ± 12.17
Female/male (n (%))	8 (66.7)/4 (33.3)
Skin-whitening cream exposure (n (%))	3 (25)
Hypertension (n (%))	3 (25)
Diabetes mellitus (n (%))	1 (8.3)
Psoriasis (n (%))	1 (8.3)
Malignancy (n (%))	2 (16.7)
Systemic lupus erythematosus (n (%))	1 (8.3)
Proteinuria in g/day (mean ± SD)	5.67 ± 4.3
Serum albumin in g/dL (mean ± SD)	2.65 ± 0.97
Serum creatinine in mg/dL (mean ± SD)	0.76 ± 0.22
Serum cholesterol (mean ± SD)	249.8 ± 100.4

Edema was the most common clinical presentation, noted in all patients; four patients (33.3%) had gross edema at presentation. Seven patients (58.3%) initially presented with nephrotic syndrome, with a mean 24-hour urine protein of 7.6 ± 4.8 g/day and a mean serum albumin of 2.1 ± 0.6 g/dL. Microscopic hematuria was present in two patients (16.7%). The mean eGFR at presentation was 109.5 ± 18.6 mL/min/1.73 m² (range: 77-130).

Kidney biopsy findings were consistent with MN in all cases. On light microscopy, all biopsies showed diffuse membranous changes with thickening of glomerular capillary walls and preserved glomerular cellularity; no segmental sclerosis was seen. Arterial changes consistent with hypertension (arteriolar medial thickening and/or intimal sclerosis) were noted in five patients, two with medial thickening and three with intimal sclerosis. Immunofluorescence demonstrated IgG1 as the dominant or co-dominant IgG subclass (along with IgG4) in 11 patients (91.7%), whereas one patient (8.3%) had IgG4-dominant deposits. C3 deposits (1+ to 2+) were present in all cases, with no IgA, IgM, IgG2, IgG3, or C1q detected. Immunohistochemistry confirmed granular capillary wall deposition of NELL-1 in all patients; none showed PLA₂R staining. Electron microscopy revealed subepithelial electron-dense deposits in all patients with extensive podocyte foot-process effacement in 10 patients (83.3%). Ten cases (83.3%) were classified as Ehrenreich and Churg stage II, characterized by the classic "spike-and-dome" appearance. The mean glomerular basement membrane thickness was 504 ± 41.8 nm.

The detailed clinical characteristics, treatments, and outcomes of individual patients are presented in Table 2.

**Table 2:** Patient characteristics, therapy, observation period, and outcome ^±^The patient was on methotrexate and apremilast. ^¥^The patient died of septic shock and respiratory failure NS: nephrotic syndrome; NIAT: nonimmunosuppressive antiproteinuric treatment; CAD: coronary artery disease; SLE: systemic lupus erythematosus; IST: immunosuppressive therapy; CR: complete remission; PR: partial remission

Patient ID	Age (years)/sex	Follow-up (months)	Clinical characteristics	Therapy type	Treatment details	Outcome (at the end of follow-up)
1	33/F	40	Ovarian teratoma, persistent NS after excision	Telmisartan, dapagliflozin; IST after 12 months	Tacrolimus, prednisone	PR
2	35/M	31	No comorbidity, NS	Ramipril; IST after 1 month	Modified Ponticelli regimen	PR
3	27/F	30	Nephrotic-range proteinuria, skin-whitening cream use	Telmisartan, cream withdrawal	No IST	CR
4	38/F	34	No comorbidity, NS	Ramipril	No IST	PR
5	50/F	17	Hypertension, subnephrotic proteinuria that worsened to NS with supportive care	Telmisartan, dapagliflozin; IST after 2 months	Tacrolimus, prednisone, rituximab	CR
6	56/F	26	No comorbidity, NS worsened on supportive care	Telmisartan; IST after 1 month	Tacrolimus, prednisone	CR
7	32/F	26	Skin-whitening cream use, subnephrotic proteinuria at presentation, initially PR with NIAT, developed NS at 1 year of supportive care	Telmisartan, dapagliflozin, cream withdrawal; IST after 12 months	Rituximab	CR
8	23/F	26	No comorbidity, subnephrotic proteinuria	Telmisartan	No IST	CR
9	60/M	5	Hypertension, smoker, NS, diagnosed with lung carcinoma after 3 months of membranous nephropathy diagnosis^¥^	Telmisartan	No IST	PR
10	40/M	14	Hypertension, diabetes, psoriatic arthritis^±^, CAD, NS	Telmisartan; IST after 1 month	Rituximab	CR
11	27/M	13	No comorbidity, subnephrotic proteinuria	Telmisartan	No IST	CR
12	26/F	13	NS, SLE with transverse myelitis, skin-whitening cream exposure	Telmisartan, upfront IST, cream withdrawal	Pulse steroids followed by taper prednisone IV cyclophosphamide	CR

Patients were followed for a mean of 22.9 ± 10.6 months. Five patients (41.7%) achieved remission with NIAT alone and did not require IST, one patient (8.3%) received upfront IST due to severe secondary disease, and the remaining six patients (50%) were started on IST after a median of 1.7 months of NIAT (range: 1-11 months). Among the five patients managed with NIAT only, one patient with underlying lung cancer died five months after presentation. The other four patients had a mean follow-up of 25.8 ± 9.1 months; by the last follow-up, three patients (60%) had achieved CR, and two (40%) had PR. Regarding time taken to achieve remission, four patients had achieved remission (PR in three and CR in one) within three months, and all five had achieved remission (PR in two and CR in three) within six months. In this NIAT-only subgroup, the mean proteinuria decreased from 3.75 ± 1.9 g/day at baseline to 0.2 ± 0.1 g/day at the last follow-up (p = 0.043), and the mean serum albumin increased from 3.04 ± 0.9 g/dL to 4.3 ± 0.1 g/dL (p = 0.043).

Across the entire cohort (excluding the one patient given upfront IST), there was no statistically significant change in proteinuria (5.7 ± 4.6 g/day at baseline vs. 4.9 ± 5.1 g/day at the last follow-up on NIAT alone; p = 0.445) or serum albumin (2.62 ± 1.02 g/dL vs. 2.95 ± 1.18 g/dL; p = 0.266) with NIAT alone. At the end of follow-up in this group, six patients (54.5%) had not achieved remission, two (18.2%) had PR, and three (27.3%) had CR. Seven patients (58.3% of the cohort) received IST at some point. Three months after the initiation of immunosuppression, the mean proteinuria decreased significantly from 8.2 ± 3.7 g/day to 3.4 ± 3.0 g/day (p = 0.018), and the mean serum albumin increased from 2.2 ± 0.6 g/dL to 3.05 ± 0.95 g/dL (p = 0.028). By three months, four patients (57.1%) in this group had achieved remission (three with PR, one with CR), while three patients (42.9%) remained without remission. At six months of IST, proteinuria further declined to 1.9 ± 1.5 g/day (p = 0.018), and the mean serum albumin rose to 3.7 ± 0.4 g/dL (p = 0.018). At that time, four patients (57.1%) had PR, two (28.6%) had CR, and one (14.3%) had no remission. After 12 months of IST, proteinuria averaged 0.64 ± 0.9 g/day (p = 0.018), and the mean serum albumin was 4.1 ± 0.3 g/dL (p = 0.018). By 12 months, all patients on immunosuppressives were in remission, five (71.4%) with CR and two (28.6%) with PR.

Overall, at the end of follow-up (mean 22.9 months), the cohort of 12 NELL-1-associated MN patients showed a significant reduction in proteinuria from 5.67 ± 4.3 g/day at baseline to 0.53 ± 0.7 g/day (p = 0.002), accompanied by an increase in mean serum albumin from 2.7 ± 0.97 g/dL to 4.04 ± 0.51 g/dL (p = 0.006). At the last follow-up, eight patients (66.7%) had achieved CR, three (25%) had PR, and one patient (8.3%), who was in PR, had died during the follow-up period.

## Discussion

NELL-1-associated MN accounted for 18.5% of all MN cases in our cohort. This proportion lies between prior reports from India (~35%) and Western populations (5-10%), with a notable female preponderance [7]. Among PLA₂R-negative MN cases in our study, NELL-1 was positive in 37.5%, aligning more closely with Western data (~23%) [7,8] than with some earlier Indian data (~83%) [7]. Differences in patient selection or population characteristics may explain this disparity.

In our study, three patients (25%) had a history of using facial skin-lightening creams. All three were young females (mean age, 29.3 years). Two patients achieved remission after discontinuing the cream and receiving supportive care alone. The third patient had coexisting SLE with transverse myelitis and required IST; she achieved CR of nephrotic syndrome within one month of treatment. Notably, her kidney biopsy did not show features of lupus nephritis (no "full house" immunofluorescence, no C1q deposits, and no tubuloreticular inclusions). We believe that, in this patient, NELL-1-associated MN was likely triggered by chronic mercury exposure from the skin cream rather than by SLE.

Mercury exposure is known to modulate the immune system and has been shown to induce autoimmunity in experimental models. Mercury-induced MN has been documented in the literature, particularly with chronic mercury exposure [9,10]. However, a definitive causal link between mercury and autoimmune disease in humans remains insufficiently proven [11,12]. Interestingly, mercury exposure has been reported to mimic the clinical and immunological features of SLE [13]. One of our cream-exposed patients initially had a PR after ceasing cream use and supportive therapy but relapsed one year later; she then achieved CR with rituximab. While NELL-1-associated MN secondary to skin-lightening creams may show a favorable initial response to exposure cessation, careful history-taking, patient education regarding risks associated with cosmetic products, and long-term follow-up are essential, as relapses may occur and additional IST may be required.

Preventive public health measures are warranted to mitigate such cases of toxin-induced MN. Regulation of over-the-counter cosmetic products, particularly skin-lightening creams, and increased public awareness about their potential harms are necessary, particularly in developing countries where lack of awareness and poor regulatory oversight contribute to hazardous exposures.

Two patients (16.7%) in our cohort had NELL-1-associated MN in the context of malignancy (one benign tumor and one lung cancer, accounting for 8.3% of the cohort). This is consistent with prior reports in which approximately 10-33% of NELL-1-associated MN cases have an associated malignancy [14]. In one of our cases, nephrotic syndrome was associated with an ovarian teratoma. Despite complete tumor resection, the patient had persistent nephrotic-range proteinuria and eventually required IST to achieve remission. This suggests that renal recovery might be delayed even after the removal of the inciting tumor antigen, possibly due to preformed immune complexes remaining in the glomeruli or ongoing immune-mediated injury independent of the tumor. These observations underscore the importance of thorough malignancy screening in MN and close follow-up even after treating a potential underlying cancer.

Half of our patients (50%) had no identified secondary cause and were considered to have primary (idiopathic) NELL-1-associated MN. These cases represented 9.2% of all MN patients and 18.7% of PLA₂R-negative MN patients in our study. This frequency aligns with previous findings by Alsharhan and Beck, who reported NELL-1 positivity in ~16% of PLA₂R-negative idiopathic MN [4]. Among our six idiopathic cases, four (66.7%) achieved CR and two (33.3%) achieved PR by the end of follow-up. Notably, half of these patients responded to supportive therapy with RAS inhibitors alone, whereas the other half required IST to attain remission.

We also report a unique association between NELL-1-positive MN and psoriatic disease. One patient in our cohort, with a long-standing history of psoriasis and psoriatic arthritis (on methotrexate and apremilast therapy), was found to have NELL-1-associated MN. To our knowledge, this is the first report linking NELL-1-positive MN with psoriatic disease. Psoriasis involves dysregulated immune pathways, which might contribute to podocyte injury or aberrant antigen presentation leading to MN. It is unclear whether the immunomodulatory therapies for psoriasis (methotrexate, apremilast) played a role in triggering MN in this patient. This case highlights the need for vigilance for kidney involvement in patients with chronic inflammatory diseases like psoriasis. Further research is needed to determine whether this represents a true pathogenic link or a coincidental finding. Larger series or mechanistic studies could clarify whether NELL-1-associated MN should be considered among the spectrum of immune-mediated renal complications of psoriatic disease.

Currently, there is no established standard therapy for NELL-1-associated MN. In general, removing an inciting agent (such as mercury) leads to a favorable outcome in many secondary cases. However, if proteinuria persists, the choice of immunosuppressive regimen is empirical, guided by treatments for primary MN and scattered case reports in the literature [15,16]. In our experience, patients responded well to therapies used for primary MN, including rituximab, calcineurin inhibitors, corticosteroids, and cyclophosphamide.

Our study has several limitations. It is a single-center study with a retrospective design, a small sample size, and no control group, which may limit the generalizability of the findings and the strength of conclusions. The retrospective nature raises the possibility of selection and information biases, and the lack of a control group prevents direct comparisons with other treatments or populations. Additionally, the follow-up duration was relatively short and may not capture long-term outcomes or relapse rates.

## Conclusions

This study provides insight into the clinical spectrum of NELL-1-associated MN, including rare associations with environmental exposures (e.g., mercury-containing skin-lightening creams), autoimmune diseases (such as psoriasis), and malignancies. The predominance of female patients, varied treatment responses, and presence of diverse secondary triggers reflect the heterogeneous nature of this entity. Notably, remission after the removal of an offending agent (when identified) underscores the importance of thorough clinical history and patient education. However, the occurrence of relapses and treatment-refractory cases highlights the need for vigilant, long-term follow-up. Given the increasing use of unregulated cosmetic products and the potential for toxin-induced immune injury, there is an urgent need for public health interventions, namely, stricter regulatory control of harmful products and public awareness campaigns, particularly in resource-limited settings.
